# The Role of Medial Frontal Cortex in Action Anticipation in Professional Badminton Players

**DOI:** 10.3389/fpsyg.2016.01817

**Published:** 2016-11-17

**Authors:** Huan Xu, Pin Wang, Zhuo’er Ye, Xin Di, Guiping Xu, Lei Mo, Huiyan Lin, Hengyi Rao, Hua Jin

**Affiliations:** ^1^Key Research Base of Humanities and Social Sciences of the Ministry of Education, Center of Cooperative Innovation for Assessment and Promotion of National Mental Health, Academy of Psychology and Behavior, Tianjin Normal UniversityTianjin, China; ^2^Guangdong Vocational College of Environmental Protection EngineeringGuangzhou, China; ^3^21cn.comGuangzhou, China; ^4^Department of Biomedical Engineering, New Jersey Institute of TechnologyNewark, USA; ^5^School of Education, Guangdong University of EducationGuangzhou, China; ^6^Center for Studies of Psychological Application, School of Psychology, South China Normal UniversityGuangzhou, China; ^7^Institute of Applied Psychology, Guangdong University of FinanceGuangzhou, China; ^8^Center for Functional Neuroimaging, Department of Neurology, University of Pennsylvania, PhiladelphiaPA, USA

**Keywords:** action anticipation, fMRI, functional connectivity, professional badminton players, medial frontal cortex

## Abstract

Some studies show that the medial frontal cortex is associated with more skilled action anticipation, while similar findings are not observed in some other studies, possibly due to the stimuli employed and the participants used as the control group. In addition, no studies have investigated whether there is any functional connectivity between the medial frontal cortex and other brain regions in more skilled action anticipation. Therefore, the present study aimed to re-investigate how the medial frontal cortex is involved in more skilled action anticipation by circumventing the limitations of previous research and to investigate that the medial frontal cortex functionally connected with other brain regions involved in action processing in more skilled action anticipation. To this end, professional badminton players and novices were asked to anticipate the landing position of the shuttlecock while watching badminton match videos or to judge the gender of the players in the matches. The video clips ended right at the point that the shuttlecock and the racket came into contact to reduce the effect of information about the trajectory of the shuttlecock. Novices who lacked training and watching experience were recruited for the control group to reduce the effect of sport-related experience on the medial frontal cortex. Blood oxygenation level-dependent activation was assessed by means of functional magnetic resonance imaging. Compared to novices, badminton players exhibited stronger activation in the left medial frontal cortex during action anticipation and greater functional connectivity between left medial frontal cortex and some other brain regions (e.g., right posterior cingulate cortex). Therefore, the present study supports the position that the medial frontal cortex plays a role in more skilled action anticipation and that there is a specific brain network for more skilled action anticipation that involves right posterior cingulate cortex, right fusiform gyrus, right inferior parietal lobule, left insula and particularly, and left medial frontal cortex.

## Introduction

From an evolutionary perspective, the anticipation of an upcoming action based on environmental cues is of great importance for individuals’ survival, as it may help individuals estimate other people’s or animal’s intentions in order to better prepare adaptive reactions to approach safety and avoid harm. Action anticipation is still important to human life, be that in daily engagement, such as driving a car or avoiding a moving object, in sports, or in combat competition. Therefore, how action anticipation influences individuals’ behavior and neural activity has been an important topic of research in psychology and human neuroscience. Expert players in interceptive sports such as badminton reacting under time pressure provide a helpful model to explore the aforementioned issue. Using the temporal occlusion paradigm, in which action is cut off at various time intervals relative to a crucial event, such as right when the shuttlecock contacts the racquet in badminton, behavioral studies have repeatedly demonstrated that compared with non-players, professional players can better predict the outcomes of other players’ sequential movements (e.g., Abernethy, 1990; Vickers, 1992; Ripoll et al., 1995; Williams and Davids, 1998; Williams and Elliott, 1999; Wolpert et al., 2003; Wilson and Knoblich, 2005; Schutz-Bosbach and Prinz, 2007; Aglioti et al., 2008; Bubic et al., 2010; Brown and Brune, 2012).

Using similar approaches, studies have also investigated whether more skilled action anticipation can be reflected in neural activity. However, the findings were mixed, particularly regarding whether the medial frontal cortex plays a role in more skilled action anticipation. Wright et al. (2010, 2011) found that compared to novices, badminton players exhibited enhanced activation in the medial frontal cortex when anticipating the direction of a badminton stroke. However, other studies did not replicate this effect. For example, Wright et al. (2013) did not find differential activation of the medial frontal cortex between high-skilled and low-skilled male soccer players when the players observed video clips of a person dribbling a ball and were required to predict the direction of the ball. Similarly, when participants were asked to predict a basketball shot’s fate, differential activation of the medial frontal cortex was not observed between athletes and novices (Abreu et al., 2012; Wu et al., 2013).

Previous studies have suggested that the medial frontal cortex plays a role in anticipation in general, when the anticipated content is unrelated to sports actions (Ridderinkhof et al., 2004; Moriguchi et al., 2007; Carrington and Bailey, 2009; Waszak et al., 2012; Corradi-Dell’Acqua et al., 2015). Therefore, activation of the medial frontal cortex should be expected during action anticipation. The fact that the studies mentioned above did not observe an effect of sports action anticipation on the medial frontal cortex may be related to the stimuli employed and the participants included in the control group. With respect to the stimuli, in previous studies, the movie clips contained information not only about the actions of the players but also about the trajectory of the ball. For example, the movie clips in Abreu et al. (2012) ended just before the ball hit the basket, fell short of it or surpassed it. Aglioti et al. (2008) found that the accuracy of action anticipation of elite players was higher than that of novices when the clip stopped right at the point the ball left the player’s hand and initiated its own trajectory; however, there were no differences between these two groups when the clips ended after the ball started its own trajectory, at the point just before the ball hit the basket. These findings suggest that the novices relied on the trajectory of the ball to perform the task. Additionally, our previous studies (Jin et al., 2010, 2011) showed that in novices, accuracy was at chance level when the clips ended right at the point that the shuttlecock contacted the racket; whereas accuracy was comparably high in this group when the shuttlecock had finished 1/3 or 2/3 of its trajectory between its point of contact with the racket and touching the ground. These findings also indicate that in novices, the ability to anticipate actions was enhanced when the clips displayed the trajectory of the shuttlecock. Showing more trajectories may allow individuals to have more cues to anticipate the outcome of the shuttlecock. This may help to enhance the accuracy of the anticipation and reduce the task difficulty for both groups, resulting in reducing group differences in the ability of anticipation and as a result, weakening the effect of more skilled action anticipation on the medial frontal cortex. Therefore, using stimuli that include the trajectory of the ball might reduce group differences in the ability of anticipation and, as a result, weaken the effect of more skilled action anticipation on the medial frontal cortex.

With regards to the participants included as the control group, while they should clearly not be professional or elite players of the related sports, in some studies, these participants were not complete “novices.” That is, the participants had some training or watching experience. For example, Wright et al. (2013) recruited low-skilled players as the control group, some of whom had previous experience that included playing for local sport clubs or school teams for more than 1 year. This experience may have enhanced the action anticipation abilities of the controls, which could reduce the differential activation of the medial frontal cortex between the players and the controls.

Therefore, the first aim of the present study was to re-investigate whether the medial frontal cortex is involved in more skilled action anticipation. To address this issue, badminton players and novice participants were presented video clips from international badminton matches and were asked to anticipate the location of the shuttlecock. To remove the effect of the trajectory of the shuttlecock, the clips ended right at the point that the shuttlecock and the racket came into contact. Participants without any training or watching experience were recruited for the control group. Based on previous studies (Wright et al., 2010, 2011), we predicted that during action anticipation, activation of the medial frontal cortex would be stronger in the badminton players compared to the novices.

In addition, cognitive task performance likely depends on connections between several brain regions. Accordingly, if the medial frontal cortex plays a role in more skilled action anticipation, this brain region may be connected more strongly with other brain regions involved in action processing, especially for more skilled action anticipation. Therefore, the second aim of the present study was to investigate how the medial frontal cortex was functionally connected with other brain regions needed for successful action anticipation. Based on evidence that experts in interceptive sports (e.g., badminton and basketball) are similar to experts in action video games, who exhibit a high level of multiple cognitive functions, including attention, executive control, and hand-eye coordination (Kida et al., 2005; Russo et al., 2006; Nakamoto and Mori, 2008a,b; Voss et al., 2010; Vestberg et al., 2012; Bejjanki et al., 2014), we predicted that expert players with more skilled action anticipation might exhibit greater functional connectivity between the medial frontal cortex and posterior regions, as observed in action video games experts during video game play (Gong et al., 2015, 2016).

## Materials and Methods

### Participants

Sixteen badminton players (14–37 years, *M* ±*SD* = 22.54 ± 5.15 years, 11 males) were recruited for the study. Players were members of professional teams or professional university teams and had completed at least 3 years of badminton training (mean = 8.81 years, range from 3 to 16 years). The control group consisted of 18 healthy novices (17–37 years, *M* ±*SD* = 21.09 ± 4.27 years, 8 males) who had no professional or amateur training in badminton or other racquet sports or no watching experience of badminton or other racquet sports. Players and novices did not differ in age (*ps.* > 0.05) or level of education. All participants were right-handed as determined by the Edinburgh Handedness Inventory (Oldfield, 1971). Participants had normal or corrected-to-normal vision and no participants had a history of neurological illness. All participants gave written informed consent prior to the study. The study was approved by School of Psychology, South China Normal University.

### Stimuli

**FIGURE 1:**
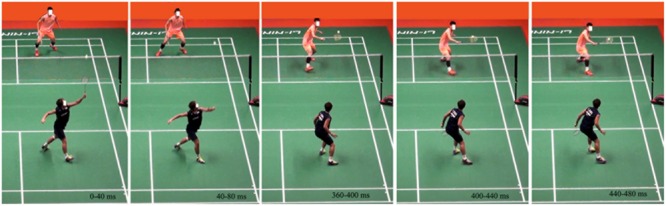
**Snapshots of a clip (480 ms in length) illustrating stimuli used in the experiment.** The clips ended at the point when the opponent’s racket touches the shuttlecock. Numbers on each frame indicate the time range of each frame (40 ms per frame). Neither the numbers nor the white square occluding the face appeared in actual experimentation.

The stimuli consisted of 40 color video clips (wmv format, 25 frames per second, including 16 practice clips) of single matches in world tournaments, as described in detail in our previous study (Jin et al., 2011, 2010). The clips depicted a player struck the shuttlecock away and an opponent receiving the shuttlecock and ended when the opponent’s racket contacted the shuttlecock. The clips did not provide any visual information about the shuttlecock’s final location or the trajectory of the shuttlecock. All of the clips lasted for 480 ms. The resolution of all the clips was 768 × 576 pixel. The resolution of the monitor was 1024 × 768 pixel with the refresh rate of 60 Hz. Examples of the stimuli were shown in **Figure 1**.

### Procedure

fMRI scanning consisted of four runs. Each run included two blocks, which varied across tasks (anticipation and gender). The sequence of the blocks was counterbalanced across the participants. Each block consisted of 20 video clips. Therefore, there were 160 trials in total (20 trials per condition × 2 conditions × 4 runs). A 20 s fixation period took place between blocks. Each block started with a 2-s instruction period. The video clips were presented for 480 ms, and the mean interval between two video clips was 3020 ms, with a range from 2520 to 3520 ms. During the presentation of the video clips, the participants were asked to pay attention to the clips. During the anticipation block, the participants were also asked to predict whether the shuttlecock would land in the forecourt or the backcourt of the players (but not the opponents’) while the clips were presented or during the following random interval. During the gender block, the participants were told to guess the gender only of the competitors. Both of the tasks emphasized speed and accuracy. Responses were given by pressing the corresponding button on a two-button pad. Response assignments were counterbalanced across participants. Stimulus presentation and the recording of behavioral responses were accomplished with E-prime software.

### Behavioral Data Recording and Analysis

For both the anticipation and the gender tasks, response accuracy and time were recorded for each video clip. Response accuracy was analyzed by one-sample test with a test value of 0.5 separately for task (anticipation versus gender) and group (player versus novice). Response accuracy and time were analysed by repeated measures analysis of variance (ANOVA) with task (anticipation versus gender) as a within-subject factor and group (player versus novice) as a between-subjects factor using SPSS 17.0 software (SPSS Inc., Chicago, IL, USA). All data are expressed as the *M ± SD* or *SE*.

### fMRI Data Acquisition and Analysis

The subjects were scanned by a Siemens 3T Trio scanner with a standard single-channel head coil. The stimuli were presented through an LCD projector onto a rear projection screen, which was located behind the participant’s head inside the magnet bore. The stimuli were presented centrally at a viewing distance of approximately 60 cm. We acquired the functional (T2*-weighted) images using blood oxygenation level-dependent (BOLD) contrast (TR = 2000 ms, TE = 30 ms, field of view = 200 × 200 mm, flip angle = 90°, matrix = 64 × 64, 32 slices/volume, in-plane resolution = 3.125 mm × 3.125 mm, no gap). For each run, the first two volumes were discarded to ensure steady-state tissue magnetization. For each participant, a T1-weighted anatomical MRI was also acquired (TE = 1.64 ms; field of view = 256 × 256 mm, flip angle = 7.0°, matrix = 256 × 256, 176 slices, voxel size = 1 mm × 1 mm × 1 mm, no gap).

Preprocessing and whole-brain analyses were performed using SPM8 (Welcome Trust Center for Neuroimaging, UCL, London, UK) implemented in Matlab 7.9. Functional connectivity analyses were conducted with the functional connectivity toolbox version 15 (CONN^1^; Whitfield-Gabrieli and Nieto-Castanon, 2012). For the pre-processing, the volumes were realigned to the first volume to minimize the effects of head movements. Then, the functional and anatomical data were co-registered. During normalization, we resampled the images at a voxel size of 3 mm × 3 mm × 3 mm and smoothed them with an 8 mm × 8 mm × 8 mm^3^ FWHM Gaussian kernel.

For the whole-brain analyses, statistical analyses based on a general linear model (GLM) were performed first at the participant level and then at the group level. At the participant level, a fixed-effects GLM was specified for each participant. Task-related changes in BOLD signal at the onset of each video clip were modeled as a delta function convolved with a haemodynamic response function (HRF). Task-related contrast was performed for each participant: anticipation > gender. The resulting contrast was then entered into separate second-level analyses, where group (player versus novice) served as a between-subjects variable in independent sample *t*-tests. The threshold in SPM was initially set to *p* < 0.001, uncorrected. Regions with *k* > 10 voxels that survived small volume correction at *p* < 0.05, FWE corrected, are reported.

In the functional connectivity analysis, a seed-based correlational analysis was used to identify the intrinsic functional connectivity of the seed, the left medial frontal cortex, across the whole brain. The left medial frontal cortex was defined based on the study by Wright et al. (2010) to avoid independent error (Vul et al., 2009). Regional time series for the seed region were extracted from bandpass-filtered images with a temporal filter (0.008–0.09 Hz). An anatomical-component-based noise correction method (aCompCor) was used to reduce noise (Behzadi et al., 2007). Global signal regression was not included to avoid potential false anti-correlations (Murphy et al., 2009). At the participant level, a GLM was used to assess significant BOLD signal correlation between the medial frontal cortex and each voxel with respect to anticipation/gender. The toolbox converted the resulting correlation coefficients to *Z*-values using Fisher transformation for subsequent *t*-tests. Differences were also examined at the group level. For the statistical parametric maps that were produced by voxel-wise analysis, clusters were considered statistically significant if they survived multiple comparisons correction. We used the approach implemented in AlphaSim^2^ based on a 3D extension of the randomization procedure described by Forman et al. (1995). The voxel-level threshold was initially set to *p* < 0.001 (uncorrected). The correction criterion was based on an estimate of the maps’ spatial smoothness and on an iterative procedure (Monte Carlo stimulation) for estimating cluster-level false-positive rates. After 1000 iterations, AlphaSim determined that an image-wide threshold of *p* < 0.05 required a cluster with 22 contiguous voxels for significance.

## Results

### Behavioral Data

The response accuracy results showed that for players, the mean accuracy for the anticipation and gender tasks was above the 0.5 chance level [*t*_(15)_ = 6.33, *p* < 0.001; *t*_(15)_ = 41.60, *p* < 0.001]. For novices, the mean accuracy for the gender task was significantly higher than 0.5 [*t*_(17)_ = 17.55, *p* < 0.001], but this was not the case for the anticipation task (*ps.* > 0.05). Additionally, the ANOVA showed main effects of task [*F*_(1,32)_ = 116.13, *p* < 0.001, η_p_^2^ = 0.78) and group (*F*_(1,32)_ = 38.70, *p*< 0.001, η_p_^2^ = 0.55). Accuracy was higher for the gender compared to the anticipation task and for players compared to novices. More importantly, the interaction between these two factors was also significant [*F*_(1,32)_ = 10.73, *p* = 0.004, η_p_^2^ = 0.23]. Further analysis showed that accuracy was higher for players compared to novices in both the anticipation [*F*_(1,32)_ = 26.77, *p* < 0.001, η_p_^2^ = 0.46] and the gender task [*F*_(1,32)_ = 16.18, *p* < 0.001, η_p_^2^ = 0.34], although to a different extent. For descriptive data, please refer to **Table 1**.

**Table 1:** Mean accuracy (%) and its standard errors (*SE*) for each experimental condition.

	Predicted task		Gender task	
	*M*	*SE*	*M*	*SE*
Experimental group	74.06	3.81	95.56	1.75
Control group	47.00	3.59	85.89	1.65

Response times were shorter for the gender compared to the anticipation task [*F*_(1,32)_ = 110.20, *p* < 0.001, η_p_^2^ = 0.78]. In the present study, limited cues were provided for the anticipation task, particularly when the trajectory of the shuttlecock was not presented. However, this seems to be not the case for the gender task. Participants could be able to identify the gender relying on some other cues (e.g., hair and clothing). Therefore, it may be easier for participants to perform the gender as compared to the anticipation task, resulting in observing shorter reaction time. In addition, while the interaction between task and group was significant [*F*_(1,32)_ = 11.78, *p* = 0.002, η_p_^2^ = 0.27], we did not find a group effect in the anticipation (*ps.* > 0.05) and gender tasks (*ps.* > 0.05). For descriptive data, please refer to **Table 2**.

**Table 2:** Mean response times (ms) and its standard errors (*SE*) for each experimental condition.

	Predicted task		Gender task	
	*M*	*SE*	*M*	*SE*
Experimental group	1135.97	67.01	699.74	32.55
Control group	970.16	63.18	748.91	30.69

### fMRI Data

#### Whole Brain Analysis

**FIGURE 2:**
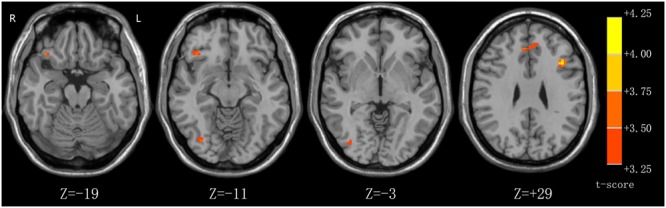
**Enhanced activations in the left medial frontal cortex to players (anticipation – gender) as compared to novices (anticipation – gender).** Statistical parametric maps are overlaid on a T1 scan.

The results of the whole-brain analysis are shown in **Table 3**. The players compared to novices showed stronger activation in the left middle frontal gyrus and left medial frontal gyrus (**Figure 2**) and in the right inferior frontal gyrus and right inferior occipital gyrus. In contrast, the novices showed no higher activation in any brain regions compared to players.

**Table 3:** Results of the whole brain analysis

Region	BA	cluster	Peak *t*	MNI coordinates		
				*X*	*Y*	*Z*
Predicted task: experimental group > control group						
Left fusiform gyrus	37	22	4.24	-42	-39	-12
			4.21	-48	-45	-9
Left middle frontal gyrus	6	35	4.22	-42	12	51
			3.56	-36	3	60
Left superior frontal gyrus	8/32	70	4.21	-18	36	48
			4.1	-24	30	57
			3.7	-15	27	39
Right medial frontal gyrus	8	42	4.16	6	36	54
			3.82	-3	33	60
			3.61	18	33	51
Right medial frontal gyrus	10/32	33	4.15	18	48	0
			3.8	6	54	12
Left inferior parietal lobule		17	3.71	-48	-39	27
**Gender task: experimental group > control group**						
Left medial frontal gyrus	10	89	4.25	-12	36	-9
**Predicted task – Gender task: experimental group > control group**						
Left middle frontal gyrus	9	20	4.25	-39	18	30
Right inferior occipital gyrus	18/19	18	4.03	36	-78	-9
			3.78	39	-81	0
Right inferior frontal gyrus	47	14	3.94	39	27	-15
Left medial frontal gyrus	8/32	22	3.68	6	36	30
			3.63	-6	42	27

#### Functional Connectivity Analysis

**FIGURE 3:**
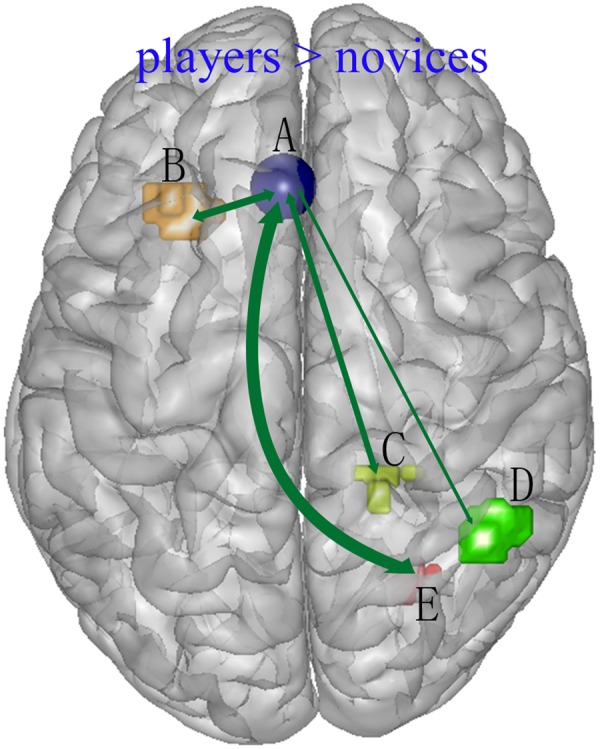
**Enhanced functional connections between left medial frontal cortex (A) and other brain regions [left insula (B), right posterior cingulate cortex (C), right inferior parietal lobule (D) and right fusiform gyrus (E)] to players (anticipation – gender) as compared to novices (anticipation – gender)**.

The brain regions that were connected with the left medial frontal cortex in relation to more skilled action anticipation are shown in **Table 4**. The left medial frontal seed region showed greater functional connectivity with the left insula and with the right posterior cingulate cortex, fusiform gyrus and inferior parietal lobule in players compared to novices (**Figure 3**). However, the novices did not show stronger connections between the left medial frontal cortex and other brain regions compared to players.

**Table 4:** Results of functional connectivity analysis.

Seed	FC Region	BA	*K*	MNI coordinates			Beta	*t*
				*X*	*Y*	*Z*		
**Experimental group > Control group:predicted task – gender task**								
Left medial frontal gyrus(BA9)	Right fusiform gyrus		108	28	-66	-10	0.24	4.66
	Left insula		67	-26	24	8	0.22	4.65
	Right inferior parietal lobule	40	63	48	-54	56	0.19	5.08
	Right posterior cingulate	29	60	18	-42	24	0.2	5.27

*FC region refer to which have functional connection with seed; K is the size of cluster; beta is the strength of connection; *p* < 0.001 (uncorrected), cluster size >40.*

## Discussion

The present study aimed to re-investigate whether more skilled action anticipation is associated with activation of the medial frontal cortex by circumventing the limitations of previous research. It also aimed to investigate how the medial frontal cortex had functional connectivity with other brain regions involved in action processing in more skilled action anticipation.

Consistent with previous studies, the behavioral results showed that response accuracy was higher for badminton players compared to novices; the novices performed the task at chance level, while this was not the case for the players (Singer et al., 1996; Abernethy et al., 2001; Rowe and McKenna, 2001; Williams et al., 2002, 2011; Cañal-Bruland et al., 2011; Jin et al., 2011). These findings demonstrate the validity of participant selection, which may be critical for successfully observing differential activation of the medial frontal cortex between the two groups.

The imaging data results showed that activation of the left medial frontal cortex was stronger in badminton players compared to novices during action anticipation, indicating that the medial frontal cortex plays a role in more skilled action anticipation. Previous studies have shown that the medial frontal cortex is involved in the anticipation of mental states (e.g., beliefs and desires; Keysers and Gazzola, 2007; Moriguchi et al., 2007; Carrington and Bailey, 2009; Corradi-Dell’Acqua et al., 2015). In a recent study by Corradi-Dell’Acqua et al. (2015), the medial orbitofrontal cortex was associated with predicting rule-based behavior. Taking previous studies and the present study together, the findings support that the left medial frontal cortex is associated with anticipation, including action anticipation.

Inconsistent with the findings of the present study, several previous studies did not find an effect of more skilled action anticipation (Abreu et al., 2012; Wright et al., 2013; Wu et al., 2013). As mentioned in the introduction, whether or not a significant effect is identified may be related to whether the participants were presented with the trajectory of the moving object and whether the participants in the control group had any experience with the related sport. In badminton, when the trajectory of the shuttlecock was presented; the nature of the anticipation became more concrete, which may be easier for participants to anticipate the location of the shuttlecock and reduce the task difficulty as a result. The simplified task may reduce the involvement of medial frontal areas for both players and novices, resulting in failing to observe the effects of skilled action anticipation. In the present study, we used clips that displayed only body kinetic information of the opponent and not information about the trajectory of the shutttlecock. We also recruited novices without any training or even watching experience. Under these conditions, we found enhanced activation of the medial frontal cortex during action anticipation in players. Therefore, information about the trajectory of the moving object and the experience of novices may be important factors that influence the effect of action anticipation.

Another important finding of the present study was that the left medial frontal cortex exhibited greater positive functional connectivity with the posterior cingulate cortex, fusiform gyrus and inferior parietal lobule in players compared to novices. These brain regions are all associated with action processing in some respect, which further suggests the involvement of the left medial frontal cortex in more skilled action anticipation. For example, the bilateral (Battelli et al., 2003) or left (Buxbaum et al., 2005; Kalénine, 2010) inferior parietal lobule was found to play a role in the recognition of actions (Battelli et al., 2003; Buxbaum et al., 2005; Kalénine, 2010). In addition, while the right or bilateral fusiform gyrus is often thought to be involved in face processing (Kanwisher et al., 1997; Duchaine and Yovel, 2015), Peelen and Downing (2005) observed that this right fusiform also plays a role in visual processing of the human body. Consistently, previous studies have shown that players were better than novices with respect to action recognition (Starkes, 1987; Abernethy et al., 1994). The posterior cingulate gyrus is thought to play an important cognitive role, and one influential hypothesis posits that the posterior cingulate cortex plays a central role in supporting internally directed cognition (Leech and Sharp, 2014). Small et al. (2003) found that right posterior cingulate cortex, together with the left medial frontal cortex, mediates the anticipatory allocation of spatial attention. Players showed a different attention pattern than non-players at key locations in a sports situation (Williams et al., 1994; Eccles et al., 2006) and paid more attention to an opponent’s body kinetic information (Williams et al., 1994; Eccles et al., 2006). Cauda et al.’s (2011) study demonstrated an important role of the bilateral insula in sensorimotor integration. In addition, Gong et al. (2015) found that action video game experts exhibited enhanced functional connectivity and gray matter volume in bilateral insular sub-regions. Similarly, badminton playing may improve players’ action processing abilities, including action recognition and visual identification of key information, as well as action anticipation; these abilities may reflect enhanced functional integration of brain regions involved in action comprehension and action anticipation.

We would like to mention several limitations of our study and suggest outlines for future research. First, the present study investigated the effect of action anticipation only by asking badminton players to predict the path of the shuttlecock; players have more skilled experience in such predictions. However, whether similar effects would be found if the players were asked to predict more general daily action sequences (e.g., inserting a spoon with food into the mouth, Reid and Striano, 2008) remains unclear. Additionally, the present study found a role of the left medial frontal cortex only in professional badminton players; it remains unclear whether this brain region would be activated in normal adults asked to predict the conclusion of common action sequences. In future studies, we may investigate these issues in greater detail. Future studies may also investigate the role of gender and experience in gender identification and action anticipation.

## Conclusion

In the present study, we observed stronger activation of the left medial frontal cortex in badminton players compared to novices during action anticipation. The left medial frontal cortex also showed stronger functional connectivity with the right cingulate and posterior cingulate cortex, right fusiform gyrus, right inferior parietal lobule and left insula in players compared to novices during action anticipation. Taken together, the findings indicate that the left medial frontal cortex is associated with more skilled action anticipation ability and that there is a specific brain network for action anticipation.

## Author Contributions

HX was involved in data analysis and manuscript drafting and revises. PW and ZY were involved in data collecting and analysis. XD and GX were involved in imaging data collecting. LM was involved in study design. HL was involved in manuscript drafting and revises. HR was involved in imaging data collecting and manuscript revises. HJ was involved in study design, execution, data analysis, and manuscript revises. We have read and approved the manuscript and agree to be accountable for all aspects of the work in ensuring that questions related to the accuracy or integrity of any part of the work are appropriately investigated and resolved.

## Conflict of Interest Statement

The authors declare that the research was conducted in the absence of any commercial or financial relationships that could be construed as a potential conflict of interest.

## Footnotes

1 www.nitrc.org/projects/conn

2 http://afni.nimh.nih.gov/afni/doc/manual/AlphaSim
